# Kentucky State Medical Society

**Published:** 1851-08

**Authors:** 


KENTUCKY STATE MEDICAL SOCIETY.

We are pleased to learn that arrangements are in progress for calling a meeting of the physicians of Kentucky, in order to organize a

State Medical Society. An organization of such a society is much needed in Kentucky, for it cannot fail to promote the welfare of the profession. We shall have more to say on the subject in our next number. B.

